# Consolidated mathematical growth model of the primary tumor and secondary distant metastases of breast cancer (CoMPaS)

**DOI:** 10.1371/journal.pone.0200148

**Published:** 2018-07-06

**Authors:** Ella Ya. Tyuryumina, Alexey A. Neznanov

**Affiliations:** International Laboratory for Intelligent Systems and Structural Analysis, Faculty of Computer science, National Research University Higher School of Economics, Moscow, Russia; University of Nebraska Medical Center, UNITED STATES

## Abstract

The goal of this research is to improve the accuracy of predicting the breast cancer (BC) process using the original mathematical model referred to as CoMPaS. The CoMPaS is the original mathematical model and the corresponding software built by modelling the natural history of the primary tumor (PT) and secondary distant metastases (MTS), it reflects the relations between the PT and MTS. The CoMPaS is based on an exponential growth model and consists of a system of determinate nonlinear and linear equations and corresponds to the TNM classification. It allows us to calculate the different growth periods of PT and MTS: 1) a non-visible period for PT, 2) a non-visible period for MTS, and 3) a visible period for MTS. The CoMPaS has been validated using 10-year and 15-year survival clinical data considering tumor stage and PT diameter. The following are calculated by CoMPaS: 1) the number of doublings for the non-visible and visible growth periods of MTS and 2) the tumor volume doubling time (days) for the non-visible and visible growth periods of MTS. The diameters of the PT and secondary distant MTS increased simultaneously. In other words, the non-visible growth period of the secondary distant MTS shrinks, leading to a decrease of the survival of patients with breast cancer. The CoMPaS correctly describes the growth of the PT for patients at the T1aN0M0, T1bN0M0, T1cN0M0, T2N0M0 and T3N0M0 stages, who does not have MTS in the lymph nodes (N0). Additionally, the CoMPaS helps to consider the appearance and evolution period of secondary distant MTS (M1). The CoMPaS correctly describes the growth period of PT corresponding to BC classification (parameter T), the growth period of secondary distant MTS and the 10-15-year survival of BC patients considering the BC stage (parameter M).

## Introduction

Breast cancer (BC) is the main cause of cancer mortality in women. BC accounts for approximately 20–25% of all the cancer types in women [1].

Finding algorithms to predict the growth of tumors has piqued the interest of researchers ever since the early days of cancer research. Several studies were carried out as an attempt to obtain reliable data on the natural history of BC growth whereas the duration of the period from the first BC cell to a patient’s death refers to the natural history of BC [2].

Mathematical modelling can play a very important role in the prognosis of the BC tumor process. Various mathematical models were built to separately describe primary tumor (PT) growth and secondary distant metastases (MTS) growth [3].

Collins et al. (1956) and Schwartz (1961) [4,5] proposed that linear exponential growth, determined by the measurement of the pulmonary MTS and expressed as *Tumor Volume Doubling Time* (TVDT), is a characteristic of individual cancers, governing the duration before and after diagnosis. Von Bertalanffy (1957) [6] built a mathematical model as an attempt to connect metabolism to growth. Laird (1964) [7,8] introduced an idea that an exact mathematical description of the tumor cell proliferation model is given by a Gompertz. Spratt et al. (1993) [9,10] determined that the BC growth rates can be calculated using a form of the logistic equation for a large group of patients undergoing routine screening mammography.

The rate of tumor growth is directly proportional to tumor size and depends on several nutrients. Since the tumor reaches a maximum size at a certain time, mathematical models, such as the Gompertz, von Bertalanffy and logistic models, describe a slowing growth rate. Consequently, these models have a common “S” shape (sigmoid curve). A sigmoid curve is a bounded, differentiable, real function that is defined for all the real input values and has a positive derivative at each point [3,6–10].

Currently, a group of classical mathematical models of PT growth includes the exponential (with or without free initial volume), Gompertz, logistic and von Bertalanffy models [11]. For breast data, the observed linear dynamics are best captured by an exponential model that is situated to describe the PT growth and the secondary distant MTS growth [12–22]. For the Gompertz and logistic models, they are used rarely in a description of PT and secondary distant MTS growth processes [23–26].

Secondary distant MTS appears at various time in different organs. The interval between the resection of PT and the first clinical manifestation of MTS (MTS free survival time or the non-visible period) is determined by the size of PT, the number of affected lymph nodes and the growth rate of MTS [12–22,24–28]. The survival (lifetime) is the period between the date of diagnosis (TNM staging system of BC) and the date of the patient’s death [1]. Survival among the BC patients (%) indicates the percentage of people in a study or treatment group who are alive for a given period after diagnosis. The percentage of patients who live at least 5, 10, 15, 20, 25 and 30 years after the PT has been treated is defined as the 5-, 10-, 15-, 20-, 25- and 30-year observed survival rate of BC patients [1,24].

Given the relation between PT and MTS, the problem of discovering the BC process seems to be two-fold. First, it is important to describe the whole natural history of BC to understand the whole process. Second, it may be necessary to predict the period of clinical MTS manifestation. However, the papers available for this do not offer other MTS mathematical growth models that relate to TNM classification. There is a call to build a mathematical model that is an exponential classical mathematical model that could describe the whole natural history of BC corresponding to TNM classification.

## Materials and methods

### Consolidated mathematical growth model of PT and secondary distant MTS (CoMPaS)

It is important to highlight that the natural history of BC continues after the resection of PT. The next stage began with the manifestation of the secondary distant MTS. When MTS reached the threshold volume, the patients died from the BC process [4,5,11–22,24].

**Fig 1:**
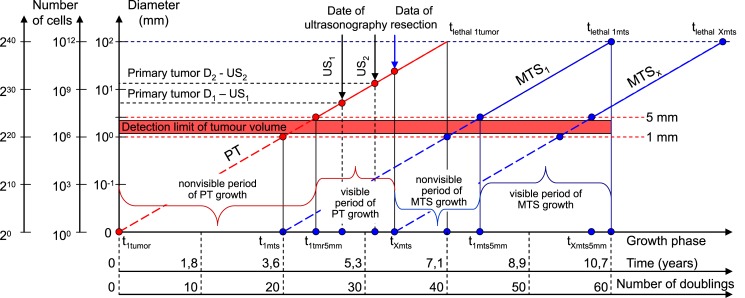
The whole natural growth history of PT and secondary distant MTS includes: The *non-visible* period of PT growth, the *visible* period of PT growth, the *non-visible* period of secondary distant MTS growth, the *visible* period of secondary distant MTS growth. Ordinate (Y): Diameter of tumor (mm). Abscissa (X): Time (years). t_1tmr_−date of appearance of the first BC stem cell; t_1tmr5mm_−the date of appearance of the visible PT with size 5 mm; t_lethal1tumor_−the date of appearance of the lethal PT with size 100 mm (when PT reaches the threshold volume); t_1mts_−the date of appearance of the first MTS cancer stem cell that coincides with the period of 20^th^ doubling time; t_1mts5mm_−the date of appearance the first visible MTS of BC with size 5 mm; t_lethal1mts_−the date of appearance the first lethal MTS of BC with size 100 mm (when secondary distant MTS reached the threshold volume); t_Xmts_−the date of appearance nXmts cell of BC MTS that coincides with date of operation; t_Xmts5mm_−the date of appearance nXmts visible BC MTS with size 5 mm; t_lethalXmts_−the date of appearance nXmts lethal BC MTS with size 100 mm; US_1_ –the date and sizes of the first US of PT; US2 –the date and sizes of the second US of PT.

All BC patients received comprehensive PT treatment. Therefore, the *whole natural history* of BC should include the period of secondary distant MTS growth (Fig 1):

1. the **non-visible** period of PT growth;
2. the **visible** period of PT growth, diagnostics and removal of PT;
3. the **non-visible** period of secondary distant MTS growth;
4. the **visible** period of secondary distant MTS growth, diagnostics, treatment and patient’s death.

The **non-visibl**e period of PT growth is from the appearance of the first tumor cell (diameter = 10 μm) until it reached a visible size (diameter = 1–5 mm).

The **visible** period of PT growth is from the point that it reached a visible size (diameter = 1–5 mm) up to the pre-surgery size.

The **non-visible** period of MTS growth can be calculated as the period from diagnosis (date of PT resection) to visible size (diameter = 1–5 mm) of at least one MTS.

The **visible** period of MTS growth can be calculated as the period from diagnosis of the visible size (diameter = 1–5 mm) to achievement of the lethal size (death).

Thus, descriptions of the *whole natural history* of BC require building a consolidated mathematical BC growth model of PT and secondary distant MTS.

It is important to define that [4,5,11–22,24,26–35]:

1. the exponential growth model was used to describe the “natural” growth rate of primary BC;
2. the "natural" rate of the secondary distant MTS is the same as the “natural” growth rate of the primary BC;
3. the period of appearance of the first metastatic cell of the secondary distant MTS coincides with the 20^th^ doubling of the primary BC. It allows us to define the non-visible growth period of MTS and the initial period of non-visible MTS manifestation;
4. the *whole nature history* of PT and secondary distant MTS is 60 doublings.

The CoMPaS may describe both the PT growth and secondary distant MTS growth considering the histopathological classification. Additionally, the CoMPaS might facilitate the survival (lifetime) and, therefore, make predictions about the future metastatic manifestation after the resection of PT.

**Fig 2:**
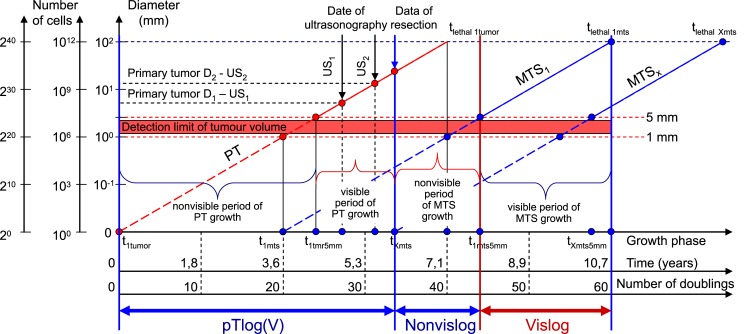
Scheme of the whole natural history of BC. The main feature is that model describes PT growth and secondary distant MTS growth as a whole (as indivisible dependent process). *pT*log (*V*)–the number of PT doublings; *Nonvislog*–the number of doublings for non-visible growth period of MTS; *Vislog*–the number of doublings for visible growth period of MTS; 60 doublings–the whole nature growth history of PT and secondary distant MTS. Tumor volume doubling time (TVDT) of PT and secondary distant MTS means a mean period (65 days).

The CoMPaS is based on an exponential growth model that consisted of nonlinear and linear deterministic equations (Fig 2) [4,5,11–22,24,26–35]:

$$\left\{ \begin{array}{c} \frac{dV}{dt}=\frac{\log2}{DT}\;V,\;t\;\leq DT\;\log_{2}\left(\frac{\theta \;DT}{\log2\;}\;V_{0}\right)\\ \frac{dV}{dt}=\theta \;\log V,\;t\;>DT\;\log_{2}\left(\frac{\theta \;DT}{\log2\;}\;V_{0}\right)\\ V\left(t=0\right)=V_{0}\\ 60=PT_{\log V\;}+Nonvis_{\log}+Vis_{\log}\\ TVDT_{non}=\;TVDT_{vis}=\;\frac{Nonvis_{days}+\;Vis_{\mathrm{days}}}{Nonvis_{\log}+Vis_{\log}} \end{array}\right.$$ (1)

where

$\frac{\log2}{DT}$ –the fraction of proliferative cells over time;

*θ* –drives the linear phase;

*PT*_log(V)_ –the number of PT doublings (Fig 2);

*Nonvis*_*log*_ –the number of doublings for the non-visible growth period of MTS (Fig 2);

*Vis*_*log*_ –the number of doublings for the visible growth period of MTS (Fig 2);

*TVDT* –tumor volume doubling time;

60 doublings –the *whole nature growth history* of PT and secondary distant MTS (Fig 2).

According to Schwartz (1961) [5], the doubling time (*DT*) can be calculated via the measurement of the tumor volume (*V*_*1*_) at surgery *t*_*1*_, the first measurement of tumor volume (*V*_*0*_) at diagnostic *t*_*0*_ and the period between the measurements (days) Δ *t* = *t*_1_ – *t*_0_:

$$DT=\frac{\Delta t\log2}{\log V_{1}-\log V_{0}}$$ (2)

where

*DT*–the doubling time period;

*V*_*1*_ –tumor volume at pre-surgery measurement time t_1_;

*V*_*0*_ –tumor volume at the time of the first measurement t_0_;

Δ *t* = *t*_1_ – *t*_0_ – the period between the first and pre-surgery measurements (days).

Friberg (1997) [20] determined that the BC tumor growth rate is constant in the visible period of tumor growth. In other words, the diameter increases from 1 mm to 100 mm [4,5,11–22,24,26–28].

The diameter of the first tumor cell equals 10 μm. After the 10^th^ doubling, the diameter of the tumor equals 100 μm (the number of cancer cells equals 10^3^). After the 20^th^ doubling, the diameter of tumor equals 1 mm (the number of cancer cells equals 10^6^), 10 mm (the number of cancer cells equals 10^9^) after the 30^th^ doubling, and 103 mm (the number of cancer cells equals 10^12^) after the 40^th^ doubling (Figs 1 and 2).

The BC PT diameter of more than 10 cm (the number of stem cells equals 10^12^ − 10^13^), is the threshold size of the tumor (lethal size). Such size promotes the BC patient’s death [4,5,11–22,24,26–28]. We may define the following three reasons that contribute to tumor growth:

1. the cycle time dividing the proliferating tumor cells;
2. the percentage of proliferating cells;
3. the percentage of spontaneous cell loss in vivo and/or migratory tumor cells.

#### Limitations

The model does not describe or explain [1,3,22]:

- the reasons for the lack of secondary MTS (M1) in patients with stage IA, IIA, IIB, (T1-4N0M0) within 25–35 years after removal of PT;
- the PT growth with MTS in the regional lymph nodes (T1-4N1-3M0) and primary MTS (T1-4N1-3M1).

### The influence of the first period were MTS appear on the life forecast for patient

A possible life forecast for the patients who have PT was designated as being:

- favourable–the appearance period of the first MTS is more than five years;
- moderately favourable–the appearance period of the first MTS is from three up to five years;
- unfavourable–the appearance period of the first MTS is less than three years.

### The study of the correlations between the non-visible growth period of the secondary distant MTS and 10-15-years survival

The mathematical models of Michaelson et al. [29–31] were used to validate the compatibility of the CoMPaS and the histopathological classification of BC with the 15-year survival correlation. According to the Michaelson et al. [29–31] equation, the data on the 15-year survival was calculated considering the PT volume (from 1 mm to 75 mm). According to the CoMPaS, the data on the non-visible MTS growth period was obtained considering the PT volume (from 1 mm to 75 mm). The correlations between the data on the 15-year survival and the data on the non-visible MTS growth period were analysed.

The mathematical models of Holzel et al. [24] and Engel et al. [32–35] was used to validate the compatibility of the CoMPaS and the histopathological classification of BC and 10-year survival correlation. According to the CoMPaS, the data on the non-visible MTS growth period was calculated considering the TNM-classification (from 7.5 mm to 47.5 mm). The correlations between the 10-year survival data and the data on the non-visible MTS growth period were analysed.

### Implementation in software

The CoMPaS was implemented as an original software application for iOS devices. It is necessary to collect predictions in one database to compare forecasts with real data and estimate the quality of the proposed model. Consequently, the CoMPaS can be connected to a database that allows us to test the model implementation. As it turns out, the new predictive tool: 1) is a solid foundation to develop future studies of BC models; 2) does not require any expensive diagnostic tests; 3) is the first predictor that makes forecasts using only current patient data, whilst the others are based on the additional statistical data.

### Statistical analysis of data

The obtained results were analysed on PC by using Python 3.5. Statistical relations between the parameters of the dataset were researched using a correlation analysis.

Statistical relations between the dataset parameters researched using a correlation analysis. The direction of the correlation was estimated by the Pearson correlation coefficient sign (r): (+)–positive and (–)–negative. The level of correlation was determined by the correlation coefficient value (r): 0 < r ≤ 0.2 –very low correlation; 0.2 < r ≤ 0.5 –low correlation; 0.5 < r ≤ 0.7 –medium correlation; 0.7 < r ≤ 0.9 –high correlation; 0.9 < r ≤ 1.0 –very high correlation. The indicator is considered significant at p < 0.05.

## Results

### Consolidated mathematical growth model of PT and secondary distant MTS of patients without lymph nodes MTS, I-II stage (CoMPaS)

Benzekry et al. (2014) [11] completed an experimental studying about the growth of the primary BC and lung cancer. As research indicates, both the Gompertz and exponential growth models describe primary BC more voraciously. Moreover, Gompertz and the exponential models describe the PT growth of BC with excellent prediction scores ≥ 80% [11]. However, the Gompertz model has some disadvantages. In particular, the model gives no opportunity a) to determine which stage the PT and secondary distant MTS are or b) to calculate the doubling time of the tumor [3,8,11,23].

The CoMPaS describes the growth of PT well and fits the BC classification (TNM). For comparing the CoMPaS and BC classification, it was first determined that the CoMPaS correctly describes all the sizes of PT in conformity with the survival of BC patients [1]. In general, the non-visible period of the secondary distant MTS growth follows the resection of PT. According to the CoMPaS, the increasing 5-year survival of BC patients depends on PT volume.

#### Calculations of the whole natural history on CoMPaS

Given all the above information, it is relevant to dwell upon building the *whole natural history* of the BC stages T1aN0M0, T1bN0M0, T1cN0M0, T2N0M0 and T3N0M0, provided that the formulas allow for calculating:

- the number of doublings for the secondary distant MTS considering only two measurements of the PT sizes;
- the correct coefficient of the secondary distant MTS spreading rate in patients without lymph node MTS related with the PT growth rate;
- the doubling time of the secondary distant MTS.

**Fig 3:**
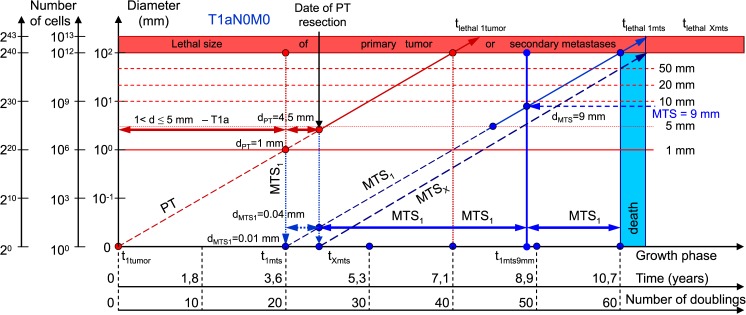
T1aN0M0. The whole natural history of PT and secondary distant MTS of patients without lymph nodes MTS via CoMPaS. Parameter T1a: 1 mm < d ≤ 5 mm. Diameter of PT at surgery of PT: d_PT_ = 4.5 mm. Diameter of secondary distant MTS at surgery of PT: d_MTS_ = 0.04 mm.

**Fig 4:**
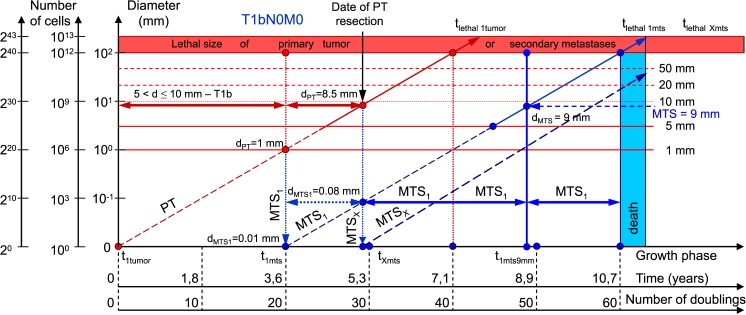
T1bN0M0. The whole natural history of PT and secondary distant MTS of patients without lymph nodes MTS via CoMPaS. Parameter T1b: 5 mm < d ≤ 10 mm. Diameter of PT at surgery of PT: d_PT_ = 8.5 mm. Diameter of secondary distant MTS at surgery of PT: d_MTS_ = 0.08 mm.

**Fig 5:**
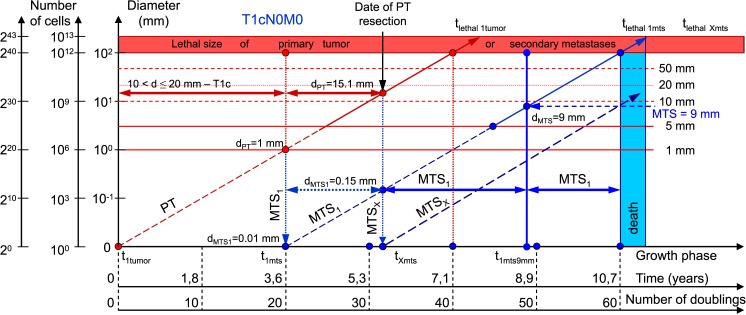
T1cN0M0. The whole natural history of PT and secondary distant MTS of patients without lymph nodes MTS via CoMPaS. Parameter T1c: 10 mm < d ≤ 20 mm. Diameter of PT at surgery of PT: d_PT_ = 15.1 mm. Diameter of secondary distant MTS at surgery of PT: d_MTS_ = 0.15 mm.

**Fig 6:**
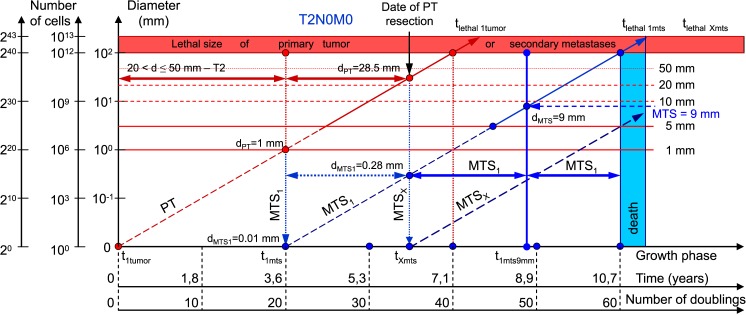
T2N0M0. The whole natural history of PT and secondary distant MTS of patients without lymph nodes MTS via CoMPaS. Parameter T2: 20 mm < d ≤ 50 mm. Diameter of PT at surgery of PT: d_PT_ = 28.5 mm. Diameter of secondary distant MTS at surgery of PT: d_MTS_ = 0.28 mm.

**Fig 7:**
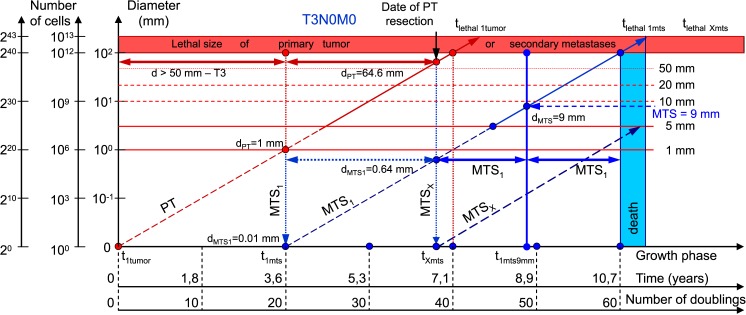
T3N0M0. The whole natural history of PT and secondary distant MTS of patients without lymph nodes MTS via CoMPaS. Parameter T3: d > 50 mm. Diameter of PT at surgery of PT: d_PT_ = 64.6 mm. Diameter of secondary distant MTS at surgery of PT: d_MTS_ = 0.64 mm.

**Fig 8:**
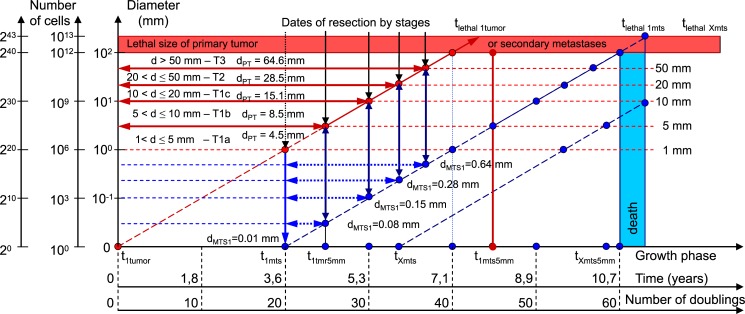
The size of secondary distant MTS. The diameter was in range of 0.08 mm and 0.64 mm at surgery date of PT (diameter below diagnostic level).

Data on the mean diameter of PT for each stage (T1, T2, T3, T4) were obtained from Table 1 of Engel et al. [33]. Table 1 and Figs 3–8 show the results of calculations via CoMPaS.

**Table 1:** The whole natural history of PT and secondary distant MTS growth of BC for different stages (pT1-4N0M0), according to CoMPaS.

	Stage T1-3N0M0				
	T1a (mm) 1 < d ≤ 5	T1b (mm) 5 < d ≤ 10	T1c (mm) 10 < d ≤ 20	T2 (mm) 20 < d ≤ 50	T3 (mm) d > 50
***pT***_***(D)***_ at surgery	**4.5**	**8.5**	**15.1**	**28.5**	**64.6**
***pT***_**log(V)**_	26.4	29.2	31.7	34.4	38.0
***TVDT***_**pT**_	70.0	70.0	70.0	65.0	60.0
***K***_**sMts(N–)**_ **(N0, n = 0)**	1.00	1.00	1.00	1.00	1.00
***TVDT***_**sMts(N–)**_ **(N0, n = 0)**	70.0	70.0	70.0	65.0	60.0
***sMTS***_**log(V)(N–)**_ **(N0, n = 0)**	6.44	9.19	11.68	14.43	17.97
***sMTS***_***D***_ **(N0, n = 0)**	0.04	0.08	0.15	0.28	0.64

*pT*_*(D)*_ at surgery (mm)–the mean size (mm) of PT at surgery (resection PT) for each stage (T1, T2, T3, T4) were obtained from Table 1 [33]; *pT*_log(V)_−the number of doublings of PT at surgery (resection PT); *TVDT*_pT_ *(days)*–the mean tumor volume doubling time of the PT at surgery (resection PT); *K*_sMts(N–)_ (N0, n = 0)–the mean correction coefficient of the MTS growth rate for N0; *TVDT*_sMts(N–)_ (N0, n = 0) *(days)*–the mean tumor volume doubling time of the secondary distant MTS for N0; *sMTS*_log(V)(N–)_ (N0, n = 0)–the number of doublings of the secondary distant MTS at surgery (resection PT) for N0; *sMTS*_*D*_ (N0, n = 0)–the mean size (mm) of the secondary distant MTS at surgery (resection PT) for N0.

The diameter of PT increases from 4.5 mm to 64.5 mm, and the diameter of the secondary distant MTS increases from 0.08 mm to 0.64 mm (Table 1; Fig 8). In general, the non-visible growth period of the secondary distant MTS continues after resection PT (Fig 8).

For the first time, the sizes of secondary distant MTS were detected in the range of 0.08 and 0.64 mm at the date of resection PT (Table 1; Fig 8). In other words, the size of the secondary distant MTS was lower than 1 mm (the minimum size for diagnostic of tumor). Consequently, secondary distant MTS could not be detected (Table 1; Fig 8). The diameter of the PT increases from 4.5 mm to 64.5 mm, and the diameter of the secondary distant MTS increases from 0.08 mm to 0.64 mm (Table 1; Fig 8).

**Fig 9:**
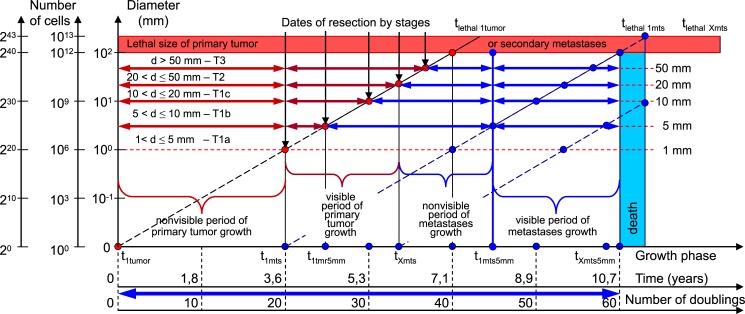
The whole natural history of PT growth. The period from the appearance of the first tumor cell up to death was in range of 9.9 and 11.5 years for pT1, pT2, pT3.

For the first time, it was determined that (Table 2; Fig 9):

- the TVDT of the MTS was in range of 60 and 70 days for pT1, pT2, and pT3 considering the sizes of the PT;
- the TVDT of the MTS was in range of 46 and 48 days for pT4. In other words, the tumor grew aggressively (pT4) considering sizes of PT;
- the *whole natural history* of the PT and MTS growth (the period from the appearance of the first tumor cell up to death) was in range of 9.9 and 11.5 years for pT1, pT2, and pT3 (Table 2; Fig 9);
- the *whole natural history* of the PT and MTS growth (the period from the appearance of the first tumor cell up to death) was in range of 7.5 and 7.9 years for pT4.

**Table 2:** The critical periods of PT and secondary distant MTS growth of BC for different stages (pT1-4N0M0), according to CoMPaS.

	Stage T1-3N0M0				
	T1a (mm) 1 < d ≤ 5	T1b (mm) 5 < d ≤ 10	T1c (mm) 10 < d ≤ 20	T2 (mm) 20 < d ≤ 50	T3 (mm) d > 50
***pT***_***(D)***_ at surgery	**4.5**	**8.5**	**15.1**	**28.5**	**64.6**
***pT***_**log(V)**_	26.4	29.2	31.7	34.4	38.0
***TVDT***_**pT**_	70.0	70.0	70.0	65.0	60.0
***WNH-PT+MTS***	11.5	11.5	11.5	10.7	9.9
***Total period PT***	5.1	5.6	6.1	6.1	6.2
***Non-visible PT***	3.8	3.8	3.8	3.5	3.3
***Visible PT***	1.2	1.8	2.3	2.6	3.0
***Non-visible MTS-I***	1.2	1.8	2.3	2.6	3.0
***Non-visible MTS-II***	4.4	3.9	3.4	2.7	1.9
***Visible MTS***	2.0	2.0	2.0	1.9	1.7
***Survival MTS***	6.4	5.9	5.4	4.5	3.6

*pT*_*(D)*_ at surgery (mm)–the mean size (mm) of PT at surgery (resection PT) for each stage (T1, T2, T3, T4) were obtained from Table 1 [33]; *pT*_log(V)_−the number of doublings of PT at surgery (resection PT); *TVDT*_pT_ *(days)*–the mean tumor volume doubling time of PT at surgery (resection PT); *WNH-PT+MTS (years)*–the whole natural history of the PT and secondary distant MTS growth for breast cancer; *Total period PT (years)*–the total period of PT growth of BC can be calculated as a period from the appearance of the first tumor cell (diameter = 10 μm) to the pre-surgery size; *Non-visible PT (years)*–the non-visible period of PT growth of BC can be calculated as a period from the appearance of the first tumor cell (diameter = 10 μm) to reaching the visible size (diameter = 1–5 mm) of PT; *Visible PT (years)*–the visible period of the PT growth for BC can be calculated as the period from the visible size (diameter = 1–5 mm) of the PT to the pre-surgery size; *Non-visible MTS-I (years)*–the non-visible period of MTS growth can be calculated as the period from the appearance of the first MTS tumor cell (diameter = 10 μm) to the non-visible size of the MTS (before date of PT surgery); *Non-visible MTS-II (years)*–the non-visible period of MTS growth can be calculated as the period from diagnosis (after date of PT surgery) to the visible size (diameter = 9 mm) of at least one MTS; *Visible MTS (years)*–the visible period of MTS growth can be calculated as the period from the diagnosis of the visible size (diameter = 9 mm) to reaching the lethal size (death); *Survival MTS (years)*–the survival (lifetime) can be calculated as the period between the date of diagnosis (TNM staging system of breast cancer) and the date of the patient’s death. The survival of BC patients included both the non-visible and visible periods of MTS growth.

The survival of BC patients included both the non-visible and visible periods of MTS growth, whereas the non-visible was the period from the date of PT resection to the date of diagnosis of secondary distant MTS (5–10), and visible was the period from the date of diagnosis of the secondary distant MTS (5–10) mm to the death of the BC patient.

For the first time, it was determined that the survival was in range of 3.6 to 6.4 years for pT1, pT2, and pT3 (Table 2; Fig 9).

For the first time, it is possible to calculate an approximate period of appearance of the first MTS cell considering the size of the PT (parameter T) for pT1, pT2, pT3.

For the first time, it is possible to calculate an approximate period of manifestation of the secondary distant MTS considering the sizes of PT (parameter T) for pT1, pT2, pT3.

For the first time, it was determined that:

- the non-visible period of MTS growth was in range of 1.9 to 4.4 years for pT1, pT2, and pT3 (Table 2; Fig 9);
- the visible period of MTS growth was in range of 1.9 to 2.0 years for pT1, pT2, and pT3 (Table 2; Fig 9);
- the visible period of MTS growth was in range of 1.8 to 1.9 years for pT4. In other words, the visible period of MTS growth for pT4 almost equalled the visible period of MTS growth for pT1, pT2, and pT3.

In 2002, Michaelson et al. [29–31] determined the high negative correlation between the 15-year survival of BC patients and the sizes of the BC PT (r = –0.98; p<0.001). In other words, the larger the size of the PT was, the lower the patient’s chance to live 15 years after PT resection was. Additionally, a high positive correlation between the probability of appearance of the secondary distant MTS and the sizes of PT were determined as (r = +0.94; p <0.001, respectively), by [29–31]. In other words, the larger the PT size of the patient was, the higher the probability of the appearance of secondary distant MTS after PT resection was.

**Fig 10:**
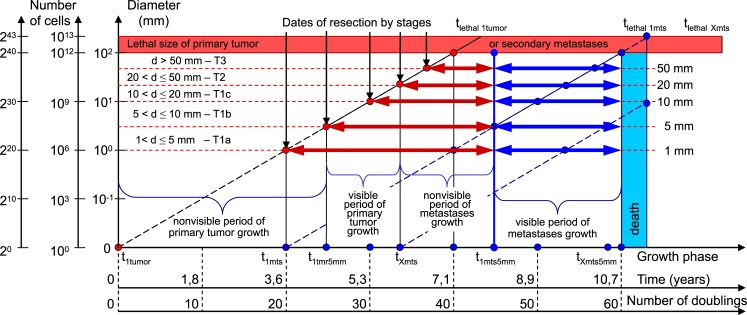
Survival includes the non-visible period (MTS free period) and the visible period of secondary distant MTS growth, diagnostics, treatment and patient’s death.

We compared data on the non-visible growth period of the secondary distant MTS and the date of the 15-year survival of the BC patients, which were obtained from the Michaelson et al. [29–31] function. We obtained a high positive correlation between the non-visible growth period and the 15-year survival of the BC patients (r = +0.94; p<0.001). In other words, the longer the non-visible growth period of the secondary distant MTS that the patient has, the higher their chance to live 15 years after PT resection (Figs 9 and 10).

In 2010, Holzel et al. [24] and Engel et al. [32–35] researched the 10- and 15-year survival from diagnosis date to PT resection on a large patient group (n = 33475) considering the BC stage (parameter T–sizes of PT). As it was demonstrated, the 15-year survival decreases with increasing stage (parameter T). However, all the differences were disappearing when patients on different stages were compared by the date of appearance of the secondary distant MTS.

We compared the data from Holzel et al. [24] and Engel et al. [32–35] on the non-visible growth period of the secondary distant MTS and the 10-year survival of BC patients. We obtained high positive correlation between the non-visible growth period of the secondary distant MTS and the 10-year survival of the BC patients (r = +0.97; p< 0.001). In other words, the longer the patient’s non-visible growth period for the secondary distant MTS, the higher their chance to live 10 years after PT resection (Figs 9 and 10).

## Discussion

In 2002, Michaelson et al. [29–31] proposed a high positive correlation between the risk of secondary distant MTS appearance in BC patients and the sizes of PT in BC (r = +0.94; p < 0.001). In other words, the larger the PT a patient has, the higher the risk the patient has for the appearance of the secondary distant MTS for 15-years after PT resection. For instance, if the size of PT equals 2 mm, then the risk of appearance of the secondary distant MTS equals 1.5% over the course of 15 years.

Michaelson et al. [29–31] determined that there was a high negative correlation between the 15-year survival of BC patients and the size of the PT (r = –0.98; p< 0.001). In other words, the larger the patient’s PT is, the higher the patient’s risk of dying 15 years after PT resection is. For instance, if the size of PT equals 2 mm, then the risk of dying for 15 years equals 1%.

In 2003, Coumans et al. [26] researched the influence of the BC stage (date of diagnosis and resection PT) on the risk of secondary distant MTS appearance using data on the BC patients (n = 38715). According to the research, the resection of a PT with a size equal to or less than 2.7 mm significantly decreases the risk of MTS appearance for 5 years (from 9.2% to 1.0%).

As can be noticed, the MTS process starts from the PT, whose size equals 1 mm. In this occasion PT contains 10^6^ tumor cells, it corresponds to the 20th doubling of PT of breast cancer. The results fit to early published data [4,5,11–22,24,26–28,32–35].

The CoMPaS model is a renewal of an exponential model of breast cancer growth [4,5,11–22,24,26–35]. Although the exponential model was implemented in different mathematical models before, the uniqueness of CoMPaS consists in the better understanding of the different clinical data by researching different growth periods [24,32–35].

The CoMPaS explains the cause of the negative correlation between the 10-year survival and the diameter of the PT in BC at the time of PT resection. The CoMPaS explains the cause of the positive correlation between the 10-year survival and the date of secondary distant MTS diagnosis [24,32–35].

The CoMPaS describes correctly the *whole natural history* of breast cancer growth. The primary tumor and secondary distant MTS growth periods contain: the non-visible growth period of PT; the visible growth period of PT; the non-visible growth period of secondary distant MTS; the visible growth period of secondary distant MTS, diagnostics, treatment and death of patients (Figs 9 and 10).

Relative survival (%) of the BC patients depends on the diameter of the PT (parameter T) (Fig 8) [24,32–35]. Survival (lifetime) is the period between the date of diagnosis (TNM staging system of BC) and the date of a patient’s death. Relative survival (%) of BC patients: 1) depends on the non-visible growth period of MTS (MTS free period); 2) does not depend on the visible growth period of MTS from the appearance of the first MTS to the date of a patient’s death (Table 2; Figs 9 and 10). The analysis of the non-visible growth period of the secondary distant MTS of BC helps to consider the cause of differences between 10-15-years of survival of the BC patients considering the BC stage (parameter M) (Figs 3–5, 9 and 10) [24,32–35].

The diameter of PT increases from 4.5 mm to 64.5 mm, and the diameter of secondary distant MTS increases from 0.08 mm to 0.64 mm (Table 1; Fig 8). This means that the non-visible period of secondary distant MTS growth decreases, which leads to a decline in the survival of the BC patients (Figs 9 and 10). Therefore, it could be concluded that the PT resection size can explain the survival time of the BC patients (Figs 9 and 10).

The analysis of the non-visible growth period of the secondary distant MTS helps to consider the cause of the differences between the 10-15-year survival of BC patients considering the BC stage (parameter M). The CoMPaS correctly estimates the survival in each group of patients who have a similar BC stage. It allows the prediction of future BC patients at the period of resection and the treatment of PT. Additionally, the CoMPaS allows us: 1) to determine the various critical growth periods of PT (the time of appearance of the first cancer stem cell, etc.) and secondary distant MTS (the time of the appearance of visible MTS, whose size is larger than 5–10 mm, etc.); and 2) to determine the groups of BC patients with high risk (during 1–5 years), medium risk (during 5–10 years), and low risk (during 10–15 years) of secondary distant MTS appearance.

As it should be highlighted, the CoMPaS can be used as a standard for the evaluation of the effectiveness of an individual treatment for each patient. If the patient lives longer with a personal treatment design, this means that the individual treatment has a positive effect. Otherwise, if a patient’s lifetime becomes shorter, the individual treatment has a negative effect. Consequently, individual treatment allows for increasing the real effectiveness for each patient.

## Conclusions

The CoMPaS allowed us to calculate: a) the number of doublings for the non-visible growth period of the secondary distant MTS; b) the number of doublings for the visible growth period of the secondary distant MTS; c) the tumor volume doubling time (days) for the non-visible growth period of the secondary distant MTS; d) the tumor volume doubling time (days) for the visible growth period of the secondary distant MTS.

A mathematical modelling of non-visible growth period of PT helps to detect the appearance period of the first tumor cell and to group more accurately the reasons for occurrence of PT. A mathematical modelling of visible growth period of PT helps to detect the appearance period of the first metastatic cell and to group more accurately the reasons for occurrence of secondary distant MTS. A mathematical modelling of non-visible growth period of secondary distant MTS helps to detect the period of diagnostic of secondary distant MTS and the period of manifestation of secondary distant MTS in different organs that may start treatment at the early stages and increase survival of patients with BC. A mathematical modelling of visible growth period of secondary distant MTS allows us to estimate a treatment effect if it increases the survival.

The CoMPaS correctly describes the growth of PT for the patients in the T1aN0M0, T1bN0M0, T1cN0M0, T2N0M0 and T3N0M0 stages, who do not have MTS in the lymph nodes (N0). Additionally, the CoMPaS helps to consider the period in which the secondary distant MTS might appear and evolve (M1).

The CoMPaS correctly describes the growth period of PT and corresponds to the BC classification (parameter T), the growth period of the secondary distant MTS and the 10-15-year survival of BC patients considering the BC stage (parameter M).

We determined via CoMPaS the variety of critical growth periods that influenced the survival forecast for BC patients. Consequently, the limits of the applicability of CoMPaS were imposed.

According to validation on the small clinical datasets, the mathematical model CoMPaS and the corresponding prediction software have a predictive capability that exceeds the predictive capability of the previous mathematical models. In further research, the pipeline includes 1) validation of the CoMPaS on a larger clinical dataset, 2) an extension of the applicability limits, and 3) software implementation in complex medical software for further oncological data analysis.
